# Microscopic Analysis of Pigments Extracted from Spalting Fungi

**DOI:** 10.3390/jof3010015

**Published:** 2017-03-14

**Authors:** Sarath M. Vega Gutierrez, Sara C. Robinson

**Affiliations:** Department of Wood Science & Engineering, 119 Richardson Hall, Oregon State University, Corvallis, OR 97331, USA; sara.robinson@oregonstate.edu

**Keywords:** extracted fungal pigments, spalting, microscopy, SEM, FIB

## Abstract

Pigments that are currently available in the market usually come from synthetic sources, or, if natural, often need mordants to bind to the target substrate. Recent research on the fungal pigment extracts from *Scytalidium cuboideum*, *Scytalidium ganodermophthorum*, *Chlorociboria aeruginosa*, and *Chlorociboria aeruginascens* have been shown to successfully dye materials, like wood, bamboo, and textiles, however, there is no information about their binding mechanisms. Due to this, a microscopic study was performed to provide information to future manufacturers interested in these pigments. The results of this study show that *S. ganodermophthorum* and *C. aeruginosa* form an amorphous layer on substrates, while *S. cuboideum* forms crystal-like structures. The attachment and morphology indicate that there might be different chemical and physical interactions between the extracted pigments and the materials. This possibility can explain the high resistance of the pigments to UV light and color fastness that makes them competitive against synthetic pigments. These properties make these pigments a viable option for an industry that demands natural pigments with the properties of the synthetic ones.

## 1. Introduction

The impact of synthetic dyes on the textile market is significant, in terms of the dyes’ ecological impact [1], and most dyes in use today come from synthetic sources. For example, the commonly used red pigments of ferrite red oxide and Venetian red contain iron oxide, cadmium, and copper oxide. One of the most common yellow pigments, “lead chromate”, and the popular green pigment, “chromium oxide”, contain the aforementioned elements [2]. Other commonly used dyes, such as aniline dyes, are amino based, and are a derivate from petroleum [3].

Natural pigment and dye alternatives do exist, although they are not competitive in the market anymore for a number of reasons. Many of these natural dyes come from plants, lichens, and insects, and may exhibit issues with color fastness [4], adherence [4], UV stability [5], and toxicity [6], among others, making them less competitive compared with their renewable counterparts. Studies on bacterial-produced pigments have promising results for their use in textiles [7], although work with fungal pigments has been disappointing. Historic fungal pigment work focused on the use of fruiting bodies of some fungal species for dye extractions, but these dyes had the same issues as other natural dyes (low resistance to UV light, need for mordants). Lichens, likewise, have similar issues [8]. However, recent research by Hinsch showed the potential of spalting fungi pigments for their application in textiles in terms of their color fastness, crocking, and stability in UV light, all without mordants [9]. Recent research on a specific group of spalting fungi, fungi that produce extracellular pigments into wood, has shown that these pigments have the potential to equal, and in some instances even outperform, synthetic pigments [10].

Pigment-type spalting fungi are a select group of soft-rotting ascomycetes that have been shown to reliably dye a number of substrates, including wood [11], bamboo [12], and textiles [9,13]. These pigments have been found to be light fast, color fast [9], and UV light stable [9,10,14]. The specific pigments of interest in these spalting fungi are as follows: *Scytalidium cuboideum* (Sacc. And Ellis) Singler and Kang, which produces a red pigment called draconin red [9]. *Scytalidium ganodermophthorum* Kand, Singler, Y.W., Lee and S.H., Yun, produces an unidentified yellow pigment [9,15], and *Chlorociboria aeruginosa* (Oeder) Seaver and *Chlorociboria aeruginascens* (Nyl.) Kanouse ex C.S. Ramamurthi, Korf and L.R. Batra. produce xylindein, a naphthoquinone blue-green pigment [16,17].

The commercial viability of the extracted pigments obtained from spalting fungi, especially if they are to be used within the textile and wood markets, requires a better understanding of their microscopic characteristics. Previous microscopic studies have focused on naturally-produced fungal pigments. Tudor et al. focused mainly in melanin producing spalting fungi in natural form, finding that the pigments located mainly in rays and fibers. Blanchette et al. used transmission electron microscopy (TEM) to verify the presence of the fungus *Chlorociboria* sp. in art pieces of the 1400s, finding evidence of hyphae mostly in rays and vessels of the wood of the analyzed art pieces [18], and Michaelsen et al. used thin-layer chromatography and mass spectrometry to identify the pigments produced by *Chlorociboria* in art pieces of the sixteenth to eighteenth century [19]. These studies have focused mainly on the pigments produced naturally by the fungi in wood. No previous works have been done on extracted fungal pigments from these specific spalting fungi under laboratory growth conditions, likely due to the fact that the pigment extraction is relatively recent [20]. Most of the pigment extraction research has been done on the genus *Chlorociboria* [21], but it was not until Robinson et al. developed a standard method to extract the pigments from the genera *Chlorociboria* and *Scytalidium* with dichloromethane (DCM) [20] that pigment extraction from spalting fungi became a common method for working with fungal pigments.

These extracted pigments have a wide spectrum of possible applications in the wood finish and textile fields. Before this can be accomplished, however, it will be necessary to better understand the spalting pigments: how they look, how they interact, and where they are deposited on their substrates. In this study, the pigments of *Chlorociboria* sp., *S. ganodermophthorum*, and *S. cuboideum*, were characterized in terms of deposition on wood and textile substrates. The information gathered from this microscopic study will give a broader understanding of how the extracted fungal pigments work, and whether or not they effectively deposit directly onto substrates, such as wood and textiles. This is important because there are no prior studies on how extracted fungal pigments deposit onto materials on a microscopic level. How dyes deposit is critical to set a basis for future research and to gain an understanding of their potential ability to bind to, and stay on, potential substrates.

## 2. Materials and Methods

Different microscopy techniques were required for the different pigments studied. All of the techniques are detailed below.

### 2.1. Light Microscopy

Fourteen-millimeter cubes of cottonwood (*Populus trichocarpa* Torr. and A. Gray) were treated with 60 drops of extracted pigments of *S. cuboideum* UAMH 11517 (isolated from *Quercus* sp*.* in Memphis, TN), *S. ganodermophthorum* UAMH 10320 (isolated from oak wood logs in Gyeonggi Province, South Korea) and *C. aeruginosa* UAMH 11657 (isolated from a decaying hardwood log in Haliburton, ON, Canada) carried in dichloromethane (DCM). All of the pigments were standardized following the values set by Robinson et al. [11,22]. The pigment drops were applied in the standard amount on the cross-section of wood blocks following the protocol of Robinson et al. [11].

After application, the blocks were air dried for 48 h in a fume hood at 20 °C to evaporate the DCM, then cut into slices between 10–14 µm with a Spencer Buffalo microtome (Spencer Lens Co., Buffalo, NY, USA). The wood slides were mounted on VWR (VWR, Radnor, PA, USA) glass slides and covered with VWR #1 glass cover slides. The imaging was done with a Nikon Eclipse Ni-U equipped with a Nikon DS-Ri2 camera (Nikon Instruments Inc., Melville, NY, USA). Samples were analyzed with special focus on the rays and vessels of the samples. Coordinates were taken from the areas that showed a higher wood coloration by the pigments for further analysis.

### 2.2. Confocal Microscopy

The samples used for the light microscopy analysis were also used for their evaluation with confocal microscopy. A Zeiss LSM 780 NLO (Carl Zeiss Microscopy, LLC, Thornwood, NY, USA) was used for this analysis. The coordinates taken during the light microscopy analysis were used on this experiment, as they were the areas of interest. Each area of interest was analyzed with a set power of the argon laser (488 nm) at 0%–2%, and the filters were set from 405 to 633 nm. The wider use of filters was used to identify the optimum combination to try to distinguish the pigments from the wood. The images were obtained with the Zeiss ZEN software and analyzed with the Zeiss ZEN 2.3 lite freeware (Carl Zeiss Microscopy).

### 2.3. Scanning Electron Microscopy (SEM)

Silica squares (2 cm × 0.5 mm) were used as a base to apply extracted pigments as a control. Fifteen drops of each fungal pigment, solubilized in DCM at a standardized concentration were applied on them [11,22]. The DCM was allowed to evaporate between each drop. Each silica piece was fixed to a Ted Pella, Inc. (Ted Pella, Inc., Redding, CA, USA) aluminum stud of one centimeter in diameter for SEM. Ted Pella, Inc. double-coated carbon conductive tape was used to fix the samples to the aluminum studs.

Wood pieces of sugar maple and cottonwood (6 mm × 3 mm × 0.3 mm) were prepared, with the wider face oriented on the radial cut. Three pieces of each were treated on a similar way than the wood blocks, using 10 and 40 drops of each extracted fungal pigment, allowing the DCM to evaporate between drops. 

Fabric squares of cotton and polyester (Testfabrics, Inc., West Pittston, PA, USA) of 5 mm × 5 mm were treated with 40 drops of extracted pigment of *S. cuboideum*, letting the DCM carrier to evaporate between drop applications. The selection of the pigment of *S. cuboideum* was done considering its characteristic morphology that would allow an easier identification of the pigments and the fibers.

The samples were mounted on Ted Pella, Inc. aluminum studs of one centimeter in diameter for SEM. A Ted Pella, Inc. double-coated carbon conductive tape was used to fix the samples to the studs. A sputter coating of gold-palladium was applied with a Cressington Sputter Coater 108 Auto (Cressington Scientific Instruments, Inc., Cranberry Twp, PA, USA) for giving the samples an enhanced optical contrast on all of the samples. The samples were exposed for 35 s to develop an even coating of 30–45 nm to avoid the electron charging of the samples and an enhanced optical contrast.

The samples were placed in the stage of an FEI QUANTA 600F environmental SEM (FEI Co., Hillsboro, OR, USA). The samples were viewed at an electron spot size of 4.5 to 5, and a high voltage (HV) between 10 to 20 kV.

### 2.4. Energy-Dispersive X-Ray Spectroscopy (EDS)

The extracted fungal pigment control samples were analyzed using the software EDAX Genesis (EDAX Inc., Mahwah, NJ, USA) for EDS analysis. For the analysis different sections of the samples were selected in the SEM and they were read using the X-ray detector. The software was calibrated for the detection of basic organic and inorganic elements. The limitation of the analysis is that it cannot detect hydrogen (H) due to its low molecular weight. For this reason, the analysis was focused on the detection of carbon (C), oxygen (O) and other elements that could have been present in the samples.

### 2.5. Focused Ion Beam (FIB)

The samples that showed higher saturation of the pigments on their surface during the SEM analysis were selected for performing an FIB cut. The selected samples were sputter coated with 10 nm of chromium oxide (Cr_2_O_3_) using a Varian Vacuum Evaporator VE10 (Agilent Technologies, Santa Clara, CA, USA). For the sputter coating, the bell of the sputter coater was opened and pieces of chromium (Cr) were placed in the holder connected to the back electrodes. On the stage below the holder, the aluminum studs with the wood samples were placed. Then the bell was closed and the vacuum system started. The first vacuum value required was 4 × 10^−1^ kPa, after reaching this value; the high-speed vacuum was activated to reach a pressure of 1 × 10^−3^ kPa. After obtaining the desired vacuum pressure, the stage where the samples were placed was set to a rotating speed of 10 rpm. After setting the vacuum and stage rotation, the electrodes where the holder with the Cr was connected was soaked at a power level of 2 for 60 s. Then, the power was increased to level 6 in 10 s. At this power level, the vaporization of the Cr and formation of the Cr_2_O_3_ started. The system remained at this level for 30 s to obtain the desired coating thickness. After 30 s, the system was turned off and the vacuum system was allowed to reach atmospheric pressure before being opened to retrieve the samples.

The coated samples were then placed in an FEI QUANTA 3D dual beam SEM/FIB (FEI Co., Hillsboro, OR, USA). Imaging of the samples was done to identify areas with higher saturation of pigments. On these areas, a rectangle of 20 µm × 3 µm was outlined. On this rectangle, two coating deposits were applied. The first coating was a layer of 20 µm × 3 µm × 100 nm of carbon (C). The second layer of coating was platinum (Pt) with the same size and depth as the carbon one. Both deposits were applied at a current of 5 Kv with 3.4 nA. After applying the deposits, the ion beam was turned on and the stage was tilted 52 degrees. The coated area was found with the ion beam and focused. There, a deposition of 1 µm of C and Pt was applied with a current of 5 Kv and 3.4 nA to protect the pre-coated area from the ion beam. Then the ion beam was activated to create a laddered cross-section of 15 µm × 1.5 µm × 5 µm at 5 Kv and 3 nA on the wood samples. The depth was enough to erode the protective coating layers, the pigment and the wood cell wall. After finishing the cross-section cutting, the borders were cleaned with the ion beam at a depth of 1 µm, to enhance the surface and clean debris generated during the cutting.

## 3. Results

### 3.1. Light Microscopy

**Figure 1:**
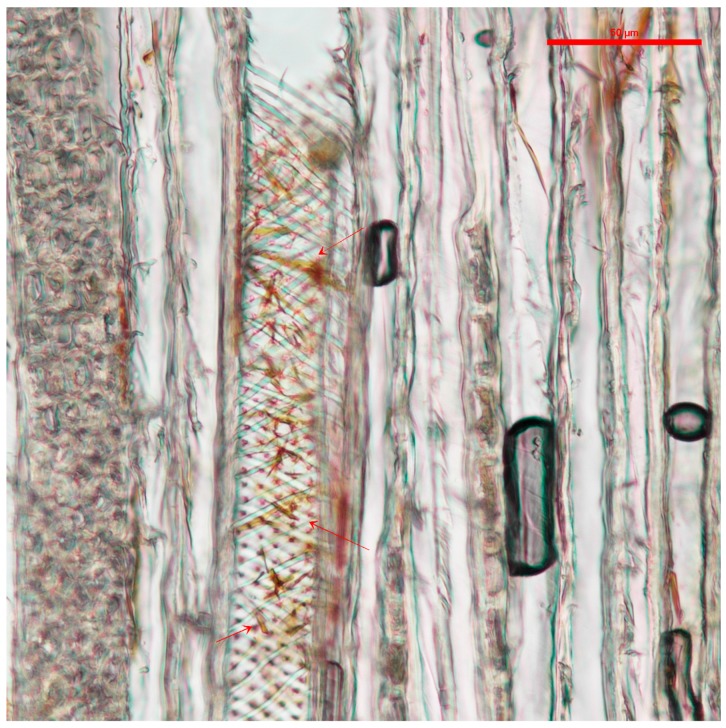
Vessel cell from cottonwood with extracted pigments of *S. cuboideum* between the helical thickenings, pointed to with arrows. Picture taken with a Nikon Eclipse Ni-U at a magnification of 20×.

The samples analyzed with light microscopy showed the pigments tended to accumulate in the wood vessels. Samples treated with *S. cuboideum* pigment extract showed filament-like structures accumulated on the vessels, especially in the helical thickenings of cottonwood, as shown in Figure 1.

**Figure 2:**
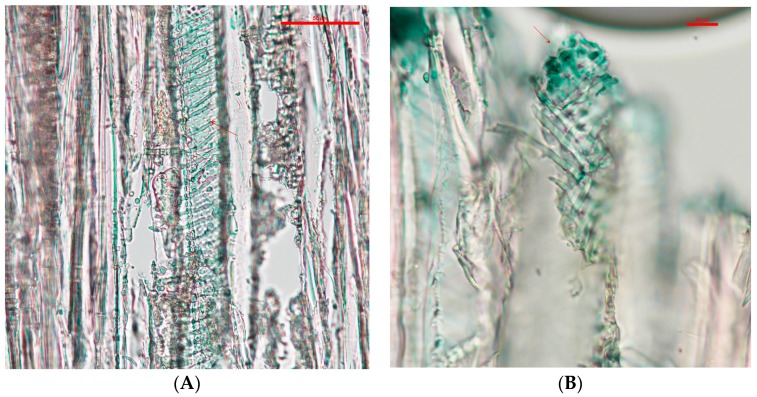
(**A**) Vessel cell wall of cottonwood covered with extracted pigment, pointed to with arrows, from *C. aeruginosa* taken at a 20× magnification; (**B**) Detail of the vessel cell wall showing concentration of the pigment in the vessel pits at a 40× magnification. Picture taken with a Nikon Eclipse Ni-U.

The wood slides that contained *C. aeruginosa* pigments tended to accumulate in the intervessel pits (see Figure 2). Compared with the pigments from *S. cuboideum*, *C. aeruginosa* pigment formed what looks like an even layer on the vessel wall and it also covered the helical thickenings.

**Figure 3:**
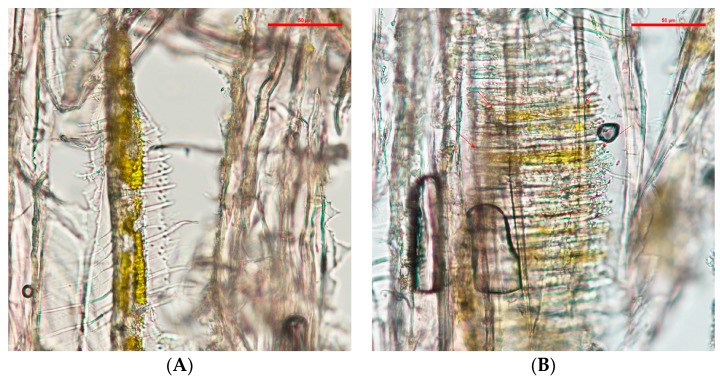
(**A**) Vessels of cottonwood with extracted pigment of *S. ganodermophthorum* accumulating on the cell walls; (**B**) Pigments of *S. ganodermophthorum* between the helical thickenings, pointed to with arrows. Picture taken with a Nikon Eclipse Ni-U at 20× magnification.

With light microscopy, the pigment of *S. ganodermophthorum* was visible on the vessel cell walls, and it also tended to accumulate on the helical thickenings (see Figure 3). Compared with the other two pigments, the yellow pigment tended to create a thicker layer on the vessel cell walls in contrast with the pigment from *C. aeruginosa*. It also accumulated between the helical thickenings, but compared to *S. cuboideum*, the pigment of *S. ganodermophthorum* did not show a characteristic shape.

### 3.2. Confocal Microscopy

With confocal microscopy, no difference between pigments and wood was visible due to the autofluorescence of wood and the broad-spectrum fluorescence of the extracted pigments at the wavelengths of 405, 488 and 561 nm. Images of the pure dry extracted pigments showed that they had textures. The red pigment had some peaks visible when analyzed at 561 nm. The yellow pigment showed an uneven texture when analyzed at 488 nm. The green pigment imaged at 488 nm showed an almost even surface. However, in general, this method showed to not be adequate for further analysis.

### 3.3. SEM on Dry Extracted Fungal Pigments

**Figure 4:**
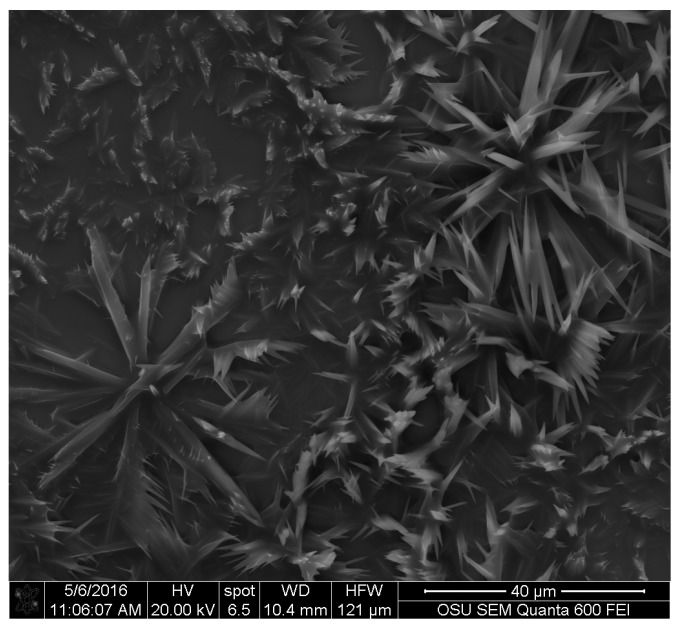
Crystal like structures from the extracted pigment of *S. cuboideum*. Picture taken with an FEI QUANTA 600 F.

Results with SEM were the most promising. Silica squares with pure *S. cuboideum* pigment showed that they crystalize and form flower-like structures, as shown in Figure 4. This structure is composed by filament-like elements, which are consistent with the results obtained with light and confocal microscopy.

**Figure 5:**
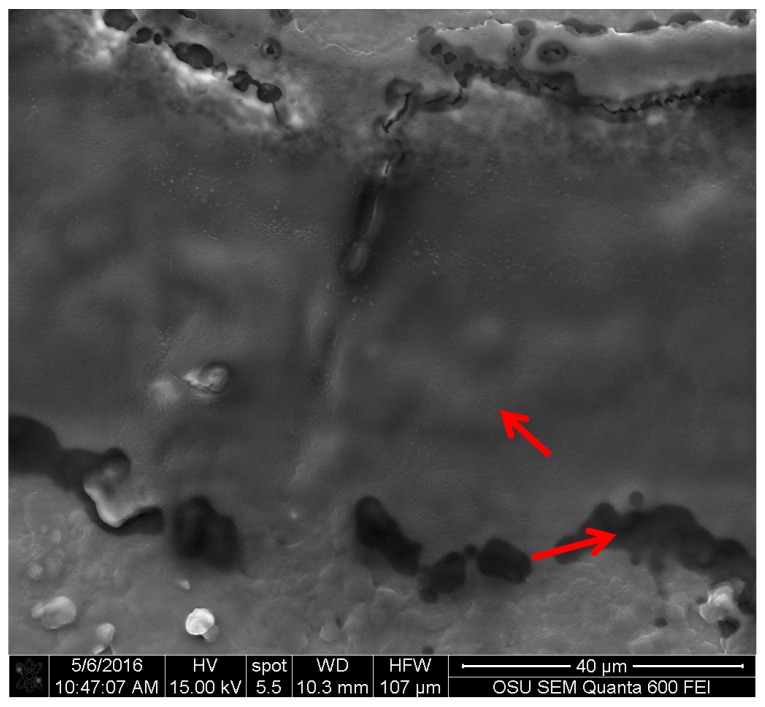
Extracted pigment of *C. aeruginosa* showing two reflective electron areas, pointed to with arrows. Picture taken with an FEI QUANTA 600 F.

The controls for *C. aeruginosa*, showed that the pigment has an amorphous structure, but it also showed two distinct areas, which can correspond to a molecular differences that affects the way that the electrons impact the compound (see Figure 5). Additionally, this pigment does not show a characteristic shape as the pigment from *S. cuboideum*, but it resembles a film.

**Figure 6:**
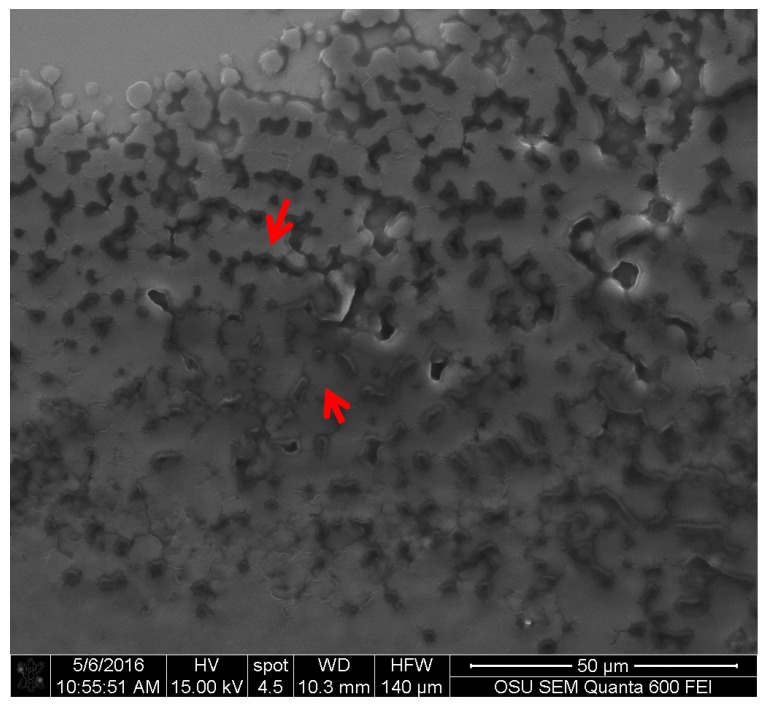
Extracted pigment of *S. ganodermophthorum* showing texture. Different electron reflectance is indicated with arrows. Picture taken with an FEI QUANTA 600 F.

The two electron reflective surface was also observed for *S. ganodermophthorum* (see Figure 6). The control also showed a more rugged surface, compared to the one of *C. aeruginosa*. This uneven surface was also observed with the light microscope and confocal microscopy, but with the use of SEM it is possible to have a more detailed view of the topography of this pigment.

### 3.4. SEM on Wood

**Figure 7:**
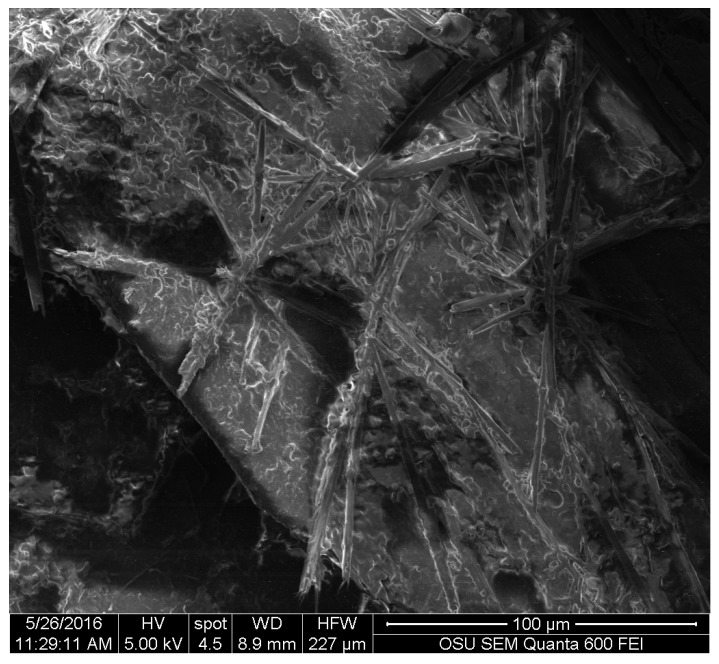
Flower-like structures formed by the extracted pigment of *S. cuboideum* on the fiber cells of cottonwood. Picture taken with an FEI QUANTA 600 F.

For the wood samples, all three pigments were deposited on the surface of wood. For *S. cuboideum*, the crystal-like structures accumulated on top of the cell walls on the radial section, and on the extremes of the cell walls. They also tended to group as seen the dry pigment on silica where the pigment forms the flower-like structure (see Figure 7) that could reach up to an average of 81 µm of diameter.

Areas with less concentration of pigment presented smaller crystals on the surface of the wood cell walls shows small crystals forming on the surface of a vessel cell wall. 

**Figure 8:**
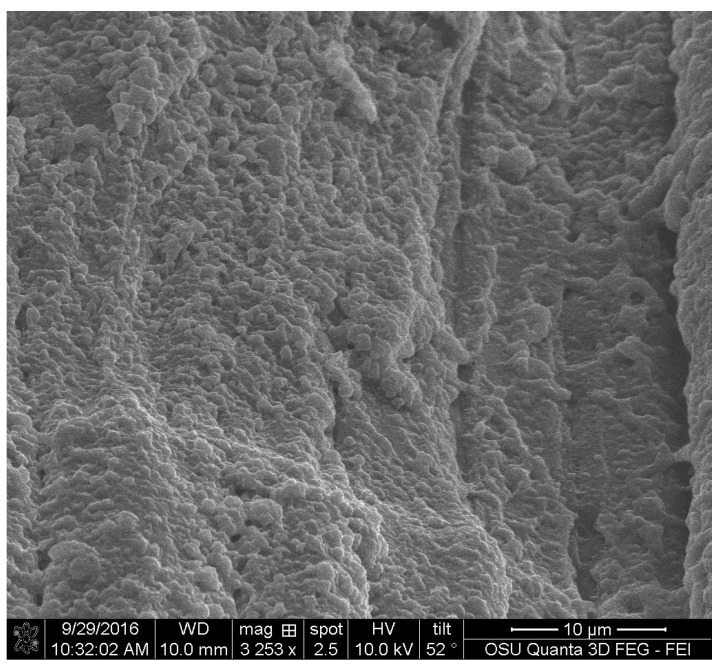
Amorphous layer formed by the extracted pigment of *C. aeruginosa*. Picture taken with an FEI QUANTA 600 F.

For *C. aeruginosa* and *S. ganodermophthorum*, the pigments formed an uneven film, covering the surface of all of the cell elements of the wood samples (see Figure 8).

### 3.5. SEM on Textiles

**Figure 9:**
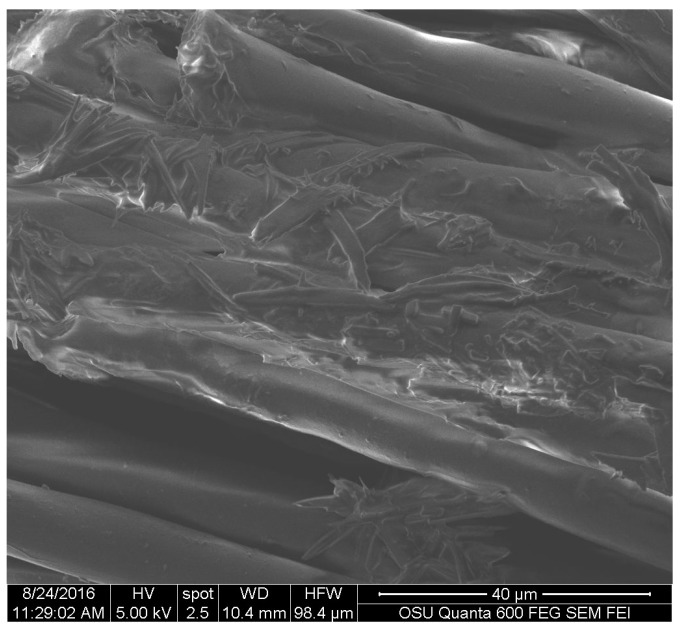
Pigment of *S. cuboideum* wrapping around polyester fibers. Picture taken with an FEI QUANTA 600 F.

The fabrics showed that the pigment of *S. cuboideum* formed crystal-like structures that wrapped to the fiber surface of the polyester sample as shown on Figure 9.

**Figure 10:**
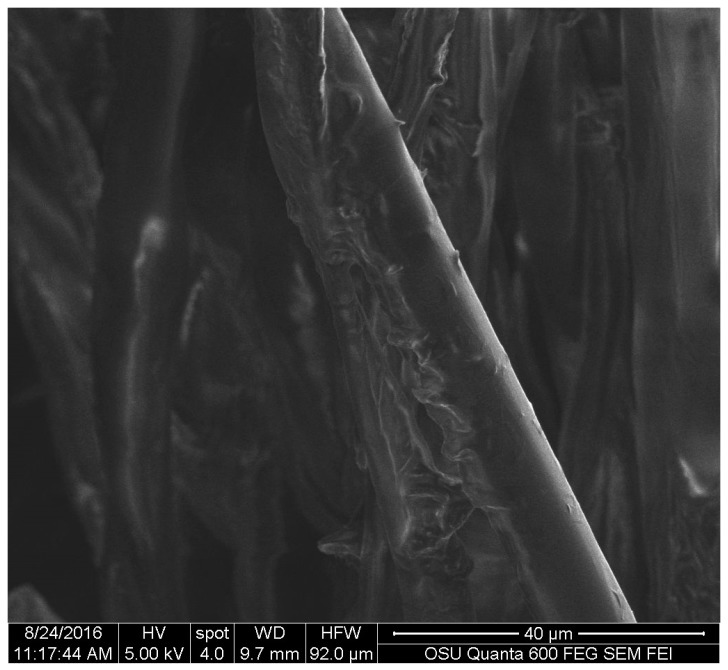
Extracted pigment of *S. cuboideum* accumulating on top of a cotton fiber. Picture taken with an FEI QUANTA 600 F.

The behavior of the pigment on cotton was similar to wood, tending to accumulate at the ends of the fibers and the pigment tended to form convoluted areas on top of the cotton fibers as shown in Figure 10.

### 3.6. EDX Analysis

The EDX analysis showed that the elements contained by the three pigments were carbon (C) and oxygen (O). No data were recorded for hydrogen (H) due to the limitation of the machine to detect that element. This made the calculation of the elements show up to 100% between C and O. In general, the pigments showed similar amounts of C and O in their structure, and we can infer the presence of hydrogen (H) on the three of them (see Table 1).

### 3.7. FIB

**Figure 11:**
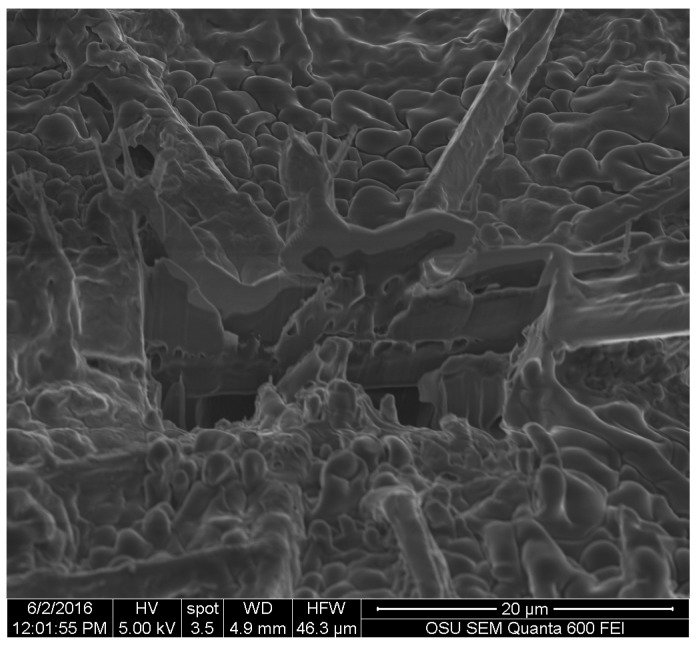
Cross-section of a crystal-like structure produced by *S. cuboideum* on cottonwood, showing void spaces between the attachment area of the pigment with the wood cell wall. Picture taken with an FEI QUANTA 3D dual beam SEM/FIB.

**Figure 12:**
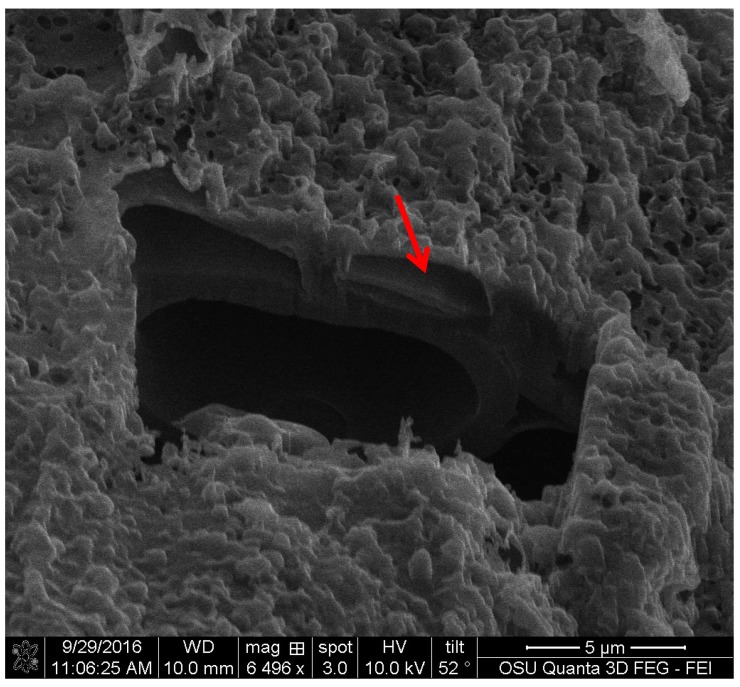
FIB cross-section of the extracted pigment of *C. aeruginosa* with cottonwood, showing large void spaces between the wood cell wall and the pigment, pointed to with an arrow. FEI QUANTA 3D dual beam SEM/FIB.

FIB images were obtained for the three pigments. They showed that all of the three extracted pigments have a surface attachment to the wood and they do not penetrate the wood cell wall. The sample of *S. cuboideum* and *C. aeruginosa* showed a similar attachment to the surface of wood, consisting of irregular void spaces between attachment points. For *S. cuboideium,* the void spaces were smaller than the ones seen for *C. aeruginosa* (see Figure 11 and Figure 12).

**Figure 13:**
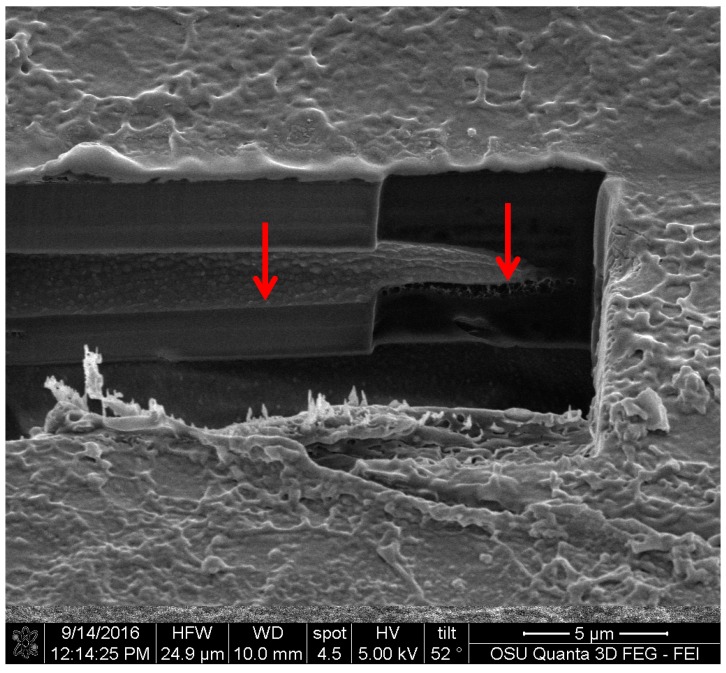
Cross-section of the area between the extracted pigment of *S. ganodermophthorum* and cottonwood. The left arrow shows an even attachment layer while the right arrow shows a sponge-like surface between the pigment and the wood. FEI QUANTA 3D dual beam SEM/FIB.

For *S. ganodermophthorum*, the attachment to wood was in two primary areas. In the first area it is possible to observe a continuous layer of pigment on top of the cell wall, and on the second area the surface showed a sponge-like texture (see Figure 13).

## 4. Discussion

The main distribution in wood of the extracted pigments was found in the vessels. The location of the extracted pigments contrast with naturally-secreted pigments. Naturally-secreted pigments will almost always be found in higher concentrations in wood ray cells, where the simple sugars are located [23]. Fungi develop and deposit their pigments in the ray cells of wood, as seen in the study by Tudor (melanin-producing fungi) [24] and Robinson [25] (pigmenting fungi, such as *C. aeruginosa*). Compared to other decay fungi, spalting fungi, specifically those that secrete extracellular pigments not classified as melanin, are Ascomycetes that prefer to first digest the available simple sugars in wood rays [23]. In contrast, extracted pigments, when applied by a dripping test, allocate in the vessels. This location is similar for wood coatings that are applied on the cross-section of wood [26], and likely has to do with the solutions moving through a path of least resistance. 

The change of location of the pigments, from rays to vessels, could affect some of the inherent properties of the spalting fungal pigments, such as UV light resistance or color fastness. Were this the case, it could be due to differences in cellular structure. Wood ray cells can compartmentalize the pigments and hold higher concentrations of them due to their septa, in contrast to the wider and continuous vessel cells [27]. Additionally, the location of the rays in the wood can make the pigments less exposed to the effects of light, as they are organized in grouped structures. This is in contrast to the alignment of vessel elements, which, depending on the wood species, can be disperse in irregular patterns. However, recent studies on extracted fungal pigments focused on textiles [9,13] and UV light resistance [14], have shown that these pigments still retain the same properties as the ones naturally deposited by fungi. 

Unfortunately the confocal microscopy method was ineffective with the samples due to the autofluorescence of wood (due to lignin and extractives) [28,29,30,31], and also because the pigments showed a similar fluorescence range. Usually, markers, like calcofluor white, are used in confocal microscopy to highlight the structures of fungal cells [32]. In the case of the extracted fungal pigments, there are no fungal cell walls where the marker can attach. This made the use of confocal microscopy not viable for further analysis of extracted fungal pigments.

Images obtained with SEM gave a detailed view of the extracted fungal pigments’ topography. A study by Vega et al. on bamboo [12], showed that the pigments of *S. cuboideum* formed a rough surface on the hyphae cell wall, while microscopy on the genus *Chlorociboria* did not find a specific structure of the pigments within the hyphae and the colored areas [18]. The lack of a specific morphology was confirmed for the extracted pigments of *S. ganodermophthorum* and *C. aeruginosa*, but for *S. cuboideum*, crystal-like structures were observed. It was originally suspected that these crystals were calcium oxalates, which are commonly formed by fungi [33,34,35], but the EDS analysis showed no presence of calcium (Ca) in the samples. Other fungal pigments are also known to crystalize as shiraiachrome B [36], a red crystal with a perylenequinone nucleus, which differs from the described naphthoquinone composition of the red pigment draconin red from *S. cuboideum*, previously described by Golinsky et al., that only includes carbon, oxygen, and hydrogen in its structure [37]. Future studies could determine if the crystal-like structures identified on *S. cuboideum* pigments are organic single crystals or organic poly-crystals (aggrupation of single crystals) by determining if it has a diffraction pattern. This could be performed with a Kikuchi diffraction pattern analysis with a transmission electron microscope (TEM), which is broadly used for determining the crystal nature of materials [38] or with the accurate X-ray diffraction analysis [39]. If the aforementioned pigment is confirmed as a single crystal or poly-crystal, the synthesis of the pure pigment could be easier regardless of the crystal type. As a crystal compound, it would be possible to obtain a powder-like state pigment, which could allow a wider use of the pigment in diverse materials without the limitation of the solvent.

Specifically for xylindein (from *C. aeruginosa*), a compound known since the late 1800s [16,17,40,41,42], two clear areas were identified in the SEM images. These two areas correspond to a difference in the way that the material reflects electrons. A study by Saikawa et al. determined that xylindein produces two isomers, one that reflects the blue spectrum of light, while the second isomer reflects the yellow spectrum of light [40]. This characteristic in the xylindein molecule can explain the presence of the two areas where the electrons reflection differs. The images obtained from *S. ganodermophthorum* also presented a similar two-electron reflection area as *C. aeruginosa*, but the chemical identification of the yellow pigment is still not completed [9,15]. By the images obtained, it is possible to suspect that there might be two different molecules in the pigment, or possibly isomers.

For the textiles, *S. cuboideum* on polyester showed that the crystal-like structures wrap around the fibers of the material. This might confer better color stability on polyester than cotton by creating a more stable pigment deposition, which could make the pigments more resistant to physical and chemical abrasions. This was confirmed by Hinsch et al., who found that the red pigment performed better on polyester, which has a chemical structure similar to lignin, in terms of color fastness compared to cellulosic fibers, such as cotton [9]. Is possible to theorize that the pigments have a higher chemical affinity to lignin-like materials over the cellulose-like ones, mainly due to a higher amount of bonding sites on lignin-like compounds compared with the cellulose-like ones [27].

For the FIB analysis, the three pigments showed a similar attachment to wood. The pigments presented irregular attachment and void areas that looked similar to how adhesives attach to wood. It is possible that the pigments form similar hydrogen bonds to wood [27], and also mechanical interactions. Future research in this area should be able to confirm this using a nuclear magnetic resonance spectroscopy (NMR) or a proton nuclear magnetic resonance (HNMR) analysis.

## 5. Conclusions

The results obtained shed new light on how these extracted pigments deposit onto wood and textiles, and offer a view of how coating and dye manufacturers can visualize the possibilities of these fungal pigments. Extracted fungal pigments from *S. cuboideum*, *C. aeruginosa*, and *S. ganodermophthorum*, when applied on the cross-section of wood, are transported through the vessels of wood and have a surface attachment that has void and contact spaces. This may mean that there can be a chemical and mechanical interaction between the pigments and the materials. On textiles it was observed that the pigment of *S. cuboideum* wrapped around the polyester fiber, while the pigment behaved similarly on cotton as it did with wood. For base morphology, the red pigment from *S. cuboideum* forms a crystal-like structure. To confirm this, diffraction studies are needed. If a crystalline structure is confirmed, a broader use of fungal pigments can be conceived as the pigments could be turned into a powdered form that can then be applied to a wider variety of materials.

Additionally, this study determined the best methods to analyze the pigments microscopically. Scanning electron microscopy was the best method to visualize and identify the extracted fungal pigments on different materials. Light microscopy was useful to identify the allocation of the pigments in wood. Confocal microscopy was not effective to identify the attachment of pigments on wood, due to the autofluorescence of the material and the broad-range fluorescence of the pigments.

## Figures and Tables

**Table 1:** Percentage of carbon (C) and oxygen (O) present in the extracted fungal pigments.

Extracted Pigment	Carbon (C) %	Oxygen (O) %	Silica (Si) and Gold (Au) % from Coating and Substrate	Total
Draconin red from *Scytalidium cuboideum*	78%	20%	2%	100%
Yellow pigment from *Scytalidium ganodermophthorum*	79%	21%	0%	100%
Xylindein from *Chlorociboria aeruginosa*	77%	23%	0%	100%
